# Visual cortex repetitive transcranial magnetic stimulation (rTMS) reversing neurodevelopmental impairments in adolescents with major psychiatric disorders (MPDs): A cross‐species translational study

**DOI:** 10.1111/cns.14427

**Published:** 2023-09-18

**Authors:** Juan Liu, Huiling Guo, Jingyu Yang, Yao Xiao, Aoling Cai, Tongtong Zhao, Fay Y. Womer, Pengfei Zhao, Junjie Zheng, Xizhe Zhang, Jie Wang, Rongxin Zhu, Fei Wang

**Affiliations:** ^1^ Early Intervention Unit, Department of Psychiatry, Affiliated Nanjing Brain Hospital Nanjing Medical University Nanjing Jiangsu China; ^2^ Functional Brain Imaging Institute of Nanjing Medical University Nanjing China; ^3^ School of Biomedical Engineering and Informatics Nanjing Medical University Nanjing Jiangsu China; ^4^ Changzhou Second People's Hospital, Changzhou Medical Center Nanjing Medical University Changzhou Jiangsu China; ^5^ Department of Psychiatry and Behavioral Sciences Vanderbilt University Medical Center Nashville Tennessee USA; ^6^ Key Laboratory of Magnetic Resonance in Biological Systems, State Key Laboratory of Magnetic Resonance and Atomic and Molecular Physics, National Center for Magnetic Resonance in Wuhan, Wuhan Institute of Physics and Mathematics, Innovation Academy for Precision Measurement Science and Technology Chinese Academy of Sciences‐Wuhan National Laboratory for Optoelectronics Wuhan China; ^7^ University of Chinese Academy of Sciences Beijing China

**Keywords:** adolescents, cross‐species, major psychiatric disorders, neurodevelopmental impairments, repetitive transcranial magnetic stimulation

## Abstract

**Aims:**

Neurodevelopmental impairments are closely linked to the basis of adolescent major psychiatric disorders (MPDs). The visual cortex can regulate neuroplasticity throughout the brain during critical periods of neurodevelopment, which may provide a promising target for neuromodulation therapy. This cross‐species translational study examined the effects of visual cortex repetitive transcranial magnetic stimulation (rTMS) on neurodevelopmental impairments in MPDs.

**Methods:**

Visual cortex rTMS was performed in both adolescent methylazoxymethanol acetate (MAM) rats and patients with MPDs. Functional magnetic resonance imaging (fMRI) and brain tissue proteomic data in rats and fMRI and clinical symptom data in patients were analyzed.

**Results:**

The regional homogeneity (ReHo) analysis of fMRI data revealed an increase in the frontal cortex and a decrease in the posterior cortex in the MAM rats, representing the abnormal neurodevelopmental pattern in MPDs. In regard to the effects of rTMS, similar neuroimaging changes, particularly reduced frontal ReHo, were found both in MAM rats and adolescent patients, suggesting that rTMS may reverse the abnormal neurodevelopmental pattern. Proteomic analysis revealed that rTMS modulated frontal synapse‐associated proteins, which may be the underpinnings of rTMS efficacy. Furthermore, a positive relationship was observed between frontal ReHo and clinical symptoms after rTMS in patients.

**Conclusion:**

Visual cortex rTMS was proven to be an effective treatment for adolescent MPDs, and the underlying neural and molecular mechanisms were uncovered. Our study provides translational evidence for therapeutics targeting the neurodevelopmental factor in MPDs.

## Graphical abstract

We found that frontal‐posterior functional imbalance may be the neurodevelopmental basis of MPDs. We also discovered that visual cortex rTMS is able to reverse the abnormal functional neurodevelopmental pattern and that modulation of synapse‐associated proteins may be the underlying molecular mechanism of rTMS efficacy.

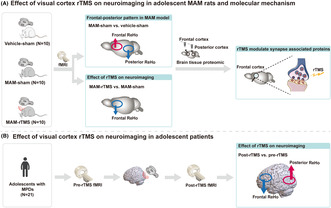

## 1 INTRODUCTION

Neurodevelopmental impairment acts as a shared etiological factor across early‐onset major psychiatric disorders (MPDs) such as schizophrenia (SZ), bipolar disorder (BD), and major depressive disorder (MDD).^1^, ^2^, ^3^, ^4^ MPDs often first appear during adolescence, which is an important period in neurodevelopment.^5^ Adolescence, characterized by heightened neuroplasticity, provides a particularly optimal interventional window to alter the developmental trajectory of illness. Therefore, targeting neurodevelopmental factor to reverse neural impairments may offer promising treatment strategies for adolescent MPDs.^6^, ^7^, ^8^

Repetitive transcranial magnetic stimulation (rTMS) as a neuromodulation technique for altering neuronal plasticity has shown to be a promising intervention for MPDs. However, in the current rTMS research for adolescent MPDs, the evidence‐based stimulation site remains uncertain.^9^ Several neurodevelopmental studies have confirmed the essential role of the primary cortex, particularly the visual cortex, in brain maturation.^10^ The spatial pattern of cortical neurodevelopment follows a posterior–anterior gradient, with the earliest maturation in the occipital visual cortex and the most protracted in superior frontal regions.^11^, ^12^ The accumulating evidence suggests that modulating the synaptic connectivity from the visual system to other brain regions may facilitate neuroplasticity in the entire brain and provide therapeutic interventions.^13^ Moreover, our previous work has shown a correlation between the frontal‐posterior functional neuroimaging pattern and neurodevelopmental factor in MPDs.^14^ In addition, abnormal neurodevelopmental trajectories have been observed to evolve through a posterior‐frontal progression from early adolescence to late adolescence, with a functional decrease in the visual cortex as the origin.^15^ In a word, the above findings converge on the idea that occipital visual cortex may be a promising rTMS target for harnessing neuroplasticity during neurodevelopmental critical periods to repair MPDs.

Animal models have proven useful in elucidating the mechanisms of rTMS as they allow us to conduct invasive molecular studies that are not ethically possible in humans.^16^, ^17^ Furthermore, animal models give us the opportunity to mitigate the confounding effects of variability that are commonly unavoidable in human studies, such as genetic background, diet, drug, and comorbidity.^18^ Hence, cross‐species studies combining human and animal samples can aid in translating preclinical findings to human research and developing novel treatment.^16^, ^19^, ^20^ The neurodevelopmental animal model, induced by administering the antimitotic agent methylazoxymethanol acetate (MAM) in pregnant rats to disrupt embryonic development during gestation day 17 (GD17), has been a widely recognized model of the neurodevelopmental factor of psychiatric disorders. MAM rats exhibit psychiatric‐like deficits of dopamine system dysfunction such as in the prefrontal cortex (PFC), which have been proven to be a core neural mechanism of MPDs across diagnostic boundaries.^21^ During adolescence, MAM rats are characterized by undifferentiated symptomatology similar to adolescent MPDs.^22^, ^23^ Therefore, the combination of the adolescent MAM model and human patients may provide a useful approach to investigate the neurodevelopmental underpinnings of MPDs.^24^ In order to bridge outcomes from animal studies and human studies, an overlapping tool with high translatability is required. Functional magnetic resonance imaging (fMRI) as a noninvasive technology for detecting brain activity, has been widely used in human studies to track neural changes of rTMS treatment.^25^ Furthermore, animal studies have also started using fMRI to understand the neural basis of MPDs because of the high translatability of this technology.^26^, ^27^ Abnormal neuroimaging patterns found in animal models mimic those observed in corresponding human studies.^28^ Thus, fMRI offers a promising noninvasive approach to align rTMS‐induced neural effects across both animal models and human samples.^20^

**FIGURE 1:**
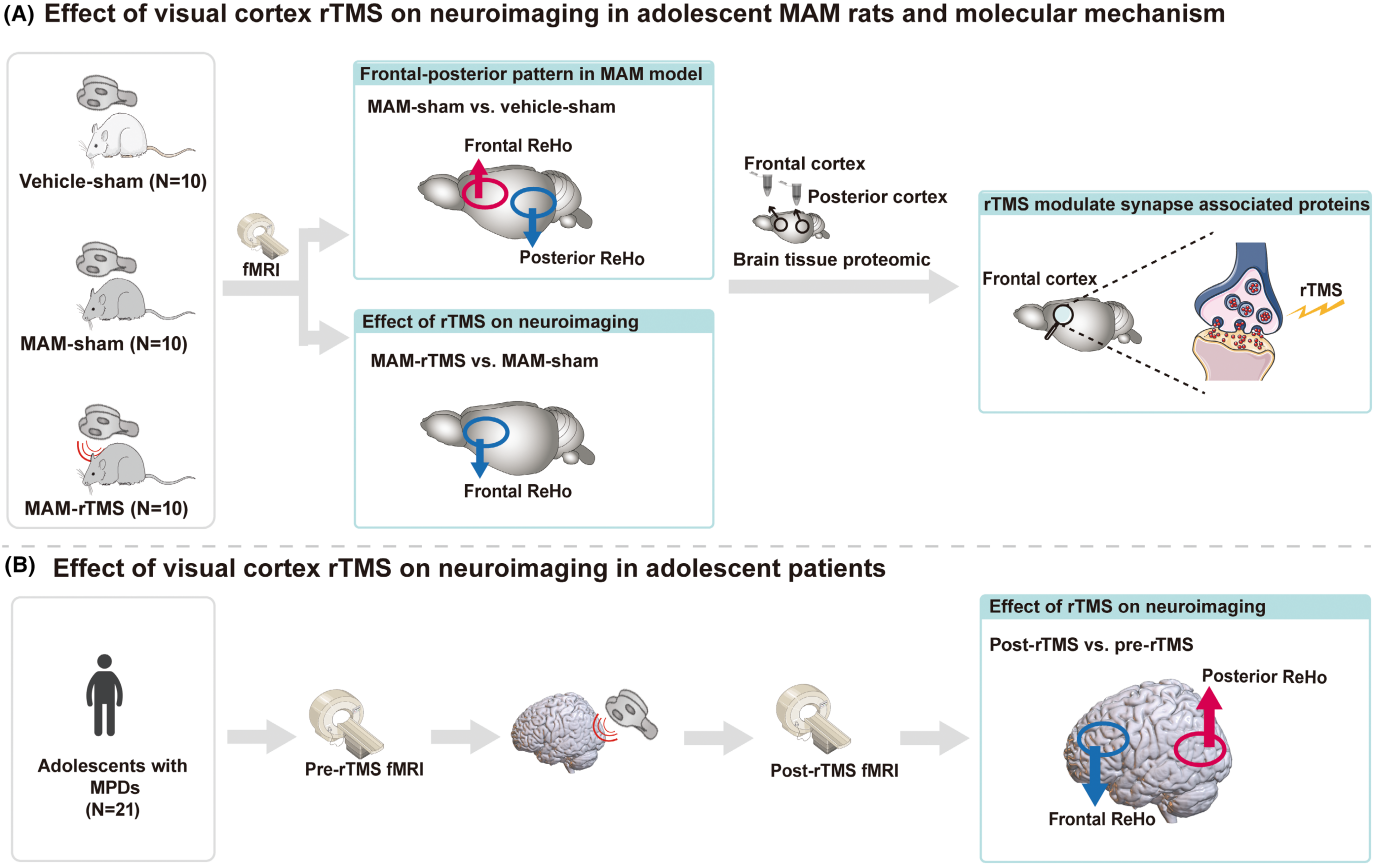
Flowchart of study design. For animal model, active/sham rTMS intervention on the visual cortex was performed during adolescence. After rTMS, neuroimaging data and brain tissue (frontal cortex and posterior cortex) proteomic data were obtained. We confirmed the frontal‐posterior functional imbalance in neurodevelopmental rat model and determined the effect of rTMS on neuroimaging and possible molecular mechanism by analyzing the rat data. Further to explore the clinical utilization, adolescent patients with MPDs received visual cortex rTMS, and neuroimaging data and clinical symptom scale data were collected before and after rTMS intervention, we confirmed the clinical efficacy of visual cortex rTMS and found a similar frontal‐posterior functional neuroimaging change of rTMS both on neurodevelopmental rat model and adolescent patients. fMRI, functional magnetic resonance imaging; MAM, methylazoxymethanol acetate; MPDs, major psychiatric disorders; ReHo, regional homogeneity; rTMS, repetitive transcranial magnetic stimulation.

Based on the above evidence, we assume that the visual cortex is a promising neuromodulation target for reversing the abnormal neurodevelopment in adolescent MPDs. We conducted a translational experiment in the present study. Visual cortex rTMS was performed in both adolescent MAM rats and adolescent patients with MPDs, changes in brain activity of rTMS were detected, and brain tissue proteomic analysis was taken to have an insight into the underpinning molecular mechanism (Figure 1).

## 2 METHODS

### 2.1 Experimental design

As shown in the study flow diagram (Figure 1), a total of 30 rats were used in the first cohort, including 20 MAM rats and 10 vehicle (vehicle‐sham) rats. MAM rats were randomly divided into MAM‐sham and MAM‐rTMS groups. Rats in the MAM‐sham and vehicle‐sham groups received sham stimulation and MAM‐rTMS rats received active rTMS intervention, with the visual cortex as the stimulation site. 2 weeks of active/sham stimulation were performed during adolescence. After stimulation, behavioral data, fMRI data and brain tissue proteomic data were obtained in the frontal cortex (referred to as frontal region‐of‐interest [ROI]) and the posterior primary sensory cortices (referred to as posterior ROI) in early adulthood. All procedures for this animal cohort complied with the Animal Research: Reporting of In Vivo Experiments (ARRIVE) guidelines and were performed in accordance with the guidelines of the Animal Care and Use Committees of the Wuhan Institute of Physics and Mathematics, Chinese Academy of Science.

In the second prospective cohort, 21 adolescents with MPDs received 10 sessions of rTMS intervention using the visual cortex as the stimulation site. Clinical symptoms were assessed and fMRI data were collected before and after rTMS. Written informed consent was obtained from all participants and their parents. The trial was approved by the Ethics Committee of the Affiliated Nanjing Brain Hospital of Nanjing Medical University and registered in the Chinese Clinical Trial Registry (ChiCTR2100045391).

### 2.2 Animals

Adult pregnant Sprague–Dawley rat dams (VITAL RIVER Laboratories, Beijing, China) were randomly injected intraperitoneally with either 22 mg/kg MAM (MAM group) or 0.9% saline solution (vehicle group) at gestational day 17.^29^ Male offspring were weaned on postnatal day (PD) 21 and utilized in our experiments. All animals were housed at a density of 2–4/cage under standard conditions with a 12 h reversed light–dark cycle (lights on 7 am, lights off 19 pm) and controlled temperature at 21 ± 1°C and humidity at 30%–70%. Furthermore, all rats had ad libitum access to both food and water.

### 2.3 Animal rTMS intervention

Prior to the stimulation protocol, rats were gently placed in a gloved hand and exposed to white noise generated by rTMS applications (5 min per day for 7 consecutive days) in order to adapt to noise and restraint during the stimulation intervention. 2 weeks of active/sham stimulation were performed during adolescence (PD 42–60). A 50 mm air‐cooled figure‐of‐eight coil was placed over the rat scalp and the coil location was the visual cortex. The stimulation scheme was as follows: 10 Hz frequency; 6 s on and 15 s off; 900 pulses per session; total duration of 5 min per session; 1 session per day for 2 weeks. For the MAM‐sham and vehicle‐sham groups, the same rTMS scheme was applied, but the coil was placed 10 cm laterally above the scalp.

### 2.4 Animal behavioral assessment

The novel object recognition (NOR) task and the open field test (OFT) were conducted in early adulthood (PD63‐75) following rTMS intervention. Detailed descriptions of the behavioral assessments can be found in Appendix S1: “Animal behavioral assessment”.

### 2.5 Animal fMRI data acquisition and processing

After anesthesia with a gas mixture of isoflurane (0.5%–1.0%), 30% O_2_ and 70% N_2_, each rat was maintained at a core temperature of 37 ± 0.5°C using a warming bed. During the fMRI acquisition, each rat was secured in a head restraint with a built‐in coil and a body tube, whose respiratory rate and body temperature were monitored using a PC‐SAM small animal monitor (SA Instruments, USA). fMRI scanning was performed on a 7.0 T MRI scanner (Bruker Biospin, Germany) equipped with a rat brain quadrature surface coil (50 mm diameter). fMRI data were acquired using an interleaved snapshot echo‐planar imaging (EPI) sequence with the following parameters: repetition time (TR) = 1000 ms, echo time (TE) = 14 ms, matrix size = 64 × 64, field of view (FOV) = 2.4 × 2.4 cm^2^, slice = 20, slice thickness = 0.8 mm, and in‐plane resolution = 375 × 375 μm. For each fMRI run, 300 frames were acquired. T1‐weighted structural scan was performed using three‐dimensional (3D) rapid imaging with a refocused echo sequence (TR of 5000 ms, TE of 12 ms, matrix size of 256 × 256, and resolution of 93.75 × 93.75 μm).

Data were preprocessed using Data Processing & Analysis of Brain Imaging (DPABI, rfmri.org/DPABI) V3.0 rat module. A standard anatomical rat template, spatially aligned to the Paxinos–Watson rat brain template, was offered and authorized.^30^ First, the voxel dimensions of both structural and functional images were scaled by a constant (10) to fit the standard neuroimaging software. Second, skull stripping was operated to the anatomical images of each rat in order to remove the MRI signal outside the brain. Third, the fMRI volumes were normalized to the standard anatomical rat template. The anatomical landmarks, including anterior commissure, fornix, posterior commissure, lateral habenular nucleus, aqueduct, paraflocculus, and pyramidal tract, were manually inspected to ensure the alignment of these landmarks between the template and studied rat, if not, the rat was discarded. Next, functional images were processed by removing the first 10 time points, slice‐timing correction, motion correction, spatial normalization, and nuisance regression with head motion parameters. After filtering with 0.01–0.08 Hz bandpass to reduce high‐frequency noise and low‐frequency drift, individual regional homogeneity (ReHo) maps were generated by calculating the Kendall's coefficient of concordance (KCC), which was used to measure the similarity of the time series for a given voxel to the neighboring 26 voxels in a 3 × 3 × 3 space. Finally, all ReHo maps were standardized using Z‐scores (zReHo) and then spatially smoothed with a 6 mm full‐width‐at‐half‐maximum (FWHM) Gaussian kernel to generate the final smoothed zReHo maps (szReHo).

### 2.6 Animal brain tissue proteomic data acquisition and processing

After behavior and fMRI data collection, rats were sacrificed on PD 73. Brains were immediately removed and rinsed in phosphate‐buffered saline (PBS). The frontal ROI and posterior ROI were located and rapidly dissected under the guidance of brain maps 4.0.^31^ The brain tissue bio‐samples were then snap‐frozen in liquid nitrogen and then stored at −80°C until proteomic analysis. A detailed procedure of proteomic investigation was provided in Appendix S1: “Animal brain tissue proteomic data acquisition and processing”.

### 2.7 Adolescent patients

Data of adolescent patients were derived from a prospective open‐label rTMS study with a single‐arm design. A total of 21 adolescent inpatients with MPDs aged 13–18 years were recruited from the Affiliated Nanjing Brain Hospital of Nanjing Medical University. All eligible participants had a diagnosis of an Axis I psychiatric disorder (including schizophrenia (*n* = 2), bipolar disorder (*n* = 12), and major depression (*n* = 7)), as assessed by the Schedule for Affective Disorders and Schizophrenia for School‐Age Children‐present and Lifetime Version (K‐SADS‐PL), based on the Diagnostic and Statistical Manual of Mental Disorders, Fourth Edition (DSM‐ IV) criteria. Exclusion criteria were as follows: major medical comorbidities (e.g., diabetes mellitus, hypertension, vascular, and infectious diseases); neurological disorders (e.g., history of head injury with loss of consciousness for ≥5 min, cerebrovascular diseases, brain tumors, and neurodegenerative diseases); unstable medical conditions (e.g., severe asthma); mental retardation or autism spectrum disorder; contraindications to MRI (e.g., severe claustrophobia, pacemakers, and metal implants); previous treatment with rTMS or electroconvulsive therapy (ECT); contraindications to rTMS (e.g., metal in the head, history of seizures); current drug/alcohol abuse or dependence. All patients were maintained on a stable psychotropic medication regimen throughout the trial. Details of demographics and baseline characteristics can be found in Table S1.

### 2.8 Patient rTMS intervention

After baseline clinical symptom assessment and fMRI scanning, all participants received 10 sessions of rTMS stimulation to visual cortex. The stimulation scheme was as follows: 10 Hz frequency; 100% of the resting motor threshold; 4 s on and 10 s off; 1200 pulses per session; total duration of 6 min 50 s per session; 2 sessions per day for 5 consecutive days. The rTMS treatment was performed using Magneuro 60 magnetic stimulator (VISHEE Inc., Nanjing, China) equipped with the figure‐of‐eight coil device. The exact location of stimulation target was determined by jointly integrating international 10–10 system and individualized 3D structural MRI. Any self‐reported adverse event was recorded after each session of treatment.

### 2.9 Patient clinical symptom assessment

The severity of clinical symptoms was assessed at baseline and after the rTMS intervention using the Brief Psychiatric Rating Scale (BPRS) to quantify general psychiatric symptoms; the Hamilton Depression Rating Scale 17‐items (HAMD) and the Hamilton Anxiety Rating Scale (HAMA) to quantify current mood symptoms.

### 2.10 Patient fMRI data acquisition and processing

fMRI scanning was performed at baseline and after rTMS intervention based on the Siemens MAGNETOM Prisma 3.0 T scanner with a standard 8‐channel head coil. The functional MRI scanning lasted for 8 min 7 s with a gradient‐echo EPI sequence, while the structural MRI scanning lasted for 5 min 58 s with a 3D magnetization‐prepared rapid acquisition gradient‐echo (MPRAGE) sequence; the parameters are as follows: Resting‐state fMRI: TR = 500 ms, TE = 30 ms, FOV = 224 **×** 224 mm^2^, matrix = 64 **×** 64, slices = 35, thickness = 3.5 mm, gap = 0.5 mm, volume = 960, voxel size = 3.5 **×** 3.5 **×** 3.5 mm^3^; 3D T1‐weighted structural images: TR = 2530 ms, TE = 2.98 ms, FOV = 224 **×** 256 mm^2^, matrix = 256 **×** 256, slices = 192, thickness = 1 mm, voxel size = 0.5 **×** 0.5 **×** 1.0 mm^3^.

Resting‐state fMRI data were processed using the Statistical Parametric Mapping 8 (SPM8; http://www.fil.ion.ucl.ac.uk/spm) and Data Processing Assistant for Resting‐State fMRI (DPARSF; http://www.restfmri.net/forum/DPARSF) toolkits.

The preprocessing procedures are as follows: removal of the first 10 time points; slice‐time correction; realignment and head‐motion correction; normalization to the standard EPI template in Montreal Neurological Institute (MNI) space and resampling to 3 **×** 3 **×** 3 mm^3^; linear detrend; nuisance regression with the Friston‐24 head motion and the polynomial trend as confounding covariates; temporal bandpass filtering (0.01–0.08 Hz). After preprocessing, individual ReHo maps were generated by calculating the KCC and then standardized to zReHo. Finally, individual szReHo maps were obtained after spatial smoothing with a 6 mm FWHM Gaussian kernel.

### 2.11 Statistical analysis

Animal behavioral data from the NOR task and OFT were analyzed by one‐way analysis of variance (ANOVA) with Bonferroni test for post hoc analysis. *p* < 0.05 was considered statistically significant.

For animal fMRI data, voxel‐based two‐sample t‐test was performed to compare ReHo between MAM‐sham group and vehicle‐sham group using DPABI. Multiple comparisons across voxels were conducted using AlphaSim correction (all corrected *p* < 0.05). As described above, we defined frontal and posterior primary sensory cortices as two ROIs in our study. Mean ReHo values were extracted from the overlapping regions between the ROIs and the different clusters identified by the previous step of the voxel‐based *t*‐test. Furthermore, one‐way ANOVA analysis was then performed among three groups, and post hoc pairwise comparisons were performed using Fisher's least significant difference (LSD) test. *p* < 0.05 was considered statistically significant.

For animal brain tissue proteomic data of frontal ROI and posterior ROI, a two‐sample t‐test was performed between MAM‐sham group and vehicle‐sham group, and between MAM‐rTMS group and MAM‐sham group, respectively. Significantly different proteins were screened according to the following criteria: fold change (FC) > 1.2 or <0.83 and uncorrected *p*‐value of the *t*‐test <0.05. Overlapping differential proteins in pairwise comparisons of MAM‐sham group and vehicle‐sham group, and of MAM‐rTMS group and MAM‐sham group, were put forward into subsequent enrichment analysis.

Enrichment analyses of the proteins in biological process (BP), cellular component (CC), and molecular function (MF) were conducted based on the Gene Ontology (GO) categorization (http://www.geneontology.org/) of each protein. GO terms with false discovery rate (FDR) corrected *p*‐value < 0.05 were considered significant.

For clinical scale data of pre‐rTMS and post‐rTMS, the normality of paired difference distribution was checked by Kolmogorov–Smirnov tests. Paired *t*‐test was applied if paired variables were normally distributed and Wilcoxon signed‐rank test was applied if not. Patient clinical scale data of BPRS, HAMD and HAMA were analyzed to detect the differences between pre‐rTMS and post‐rTMS. For fMRI data, ReHo differences between pre‐rTMS and post‐rTMS treatment were identified by whole‐brain voxel‐wise paired *t*‐test (all *p* < 0.05, AlphaSim corrected). To further establish the relationship between clinical efficacy and neuroimaging changes, correlation analyses were conducted between the scale total scores and significant ReHo values after rTMS intervention.

## 3 RESULTS

### 3.1 Behavioral abnormality in MAM rats

To clarify how the visual cortex rTMS affects the neurodevelopmental underpinnings of MPDs, we performed the treatment on MAM rats.^32^ In the behavioral data analyses, compared to vehicle‐sham rats, MAM‐sham rats showed lower discrimination index of the NOR task (*p* = 0.004, Bonferroni corrected), higher distance (*p* = 0.003, Bonferroni corrected), higher mean speed (*p* = 0.003, Bonferroni corrected) and higher central distance of the OFT test (*p* = 0.034, Bonferroni corrected), suggesting impaired learning and recognition processing, and abnormal locomotion and exploratory behavior in MAM‐sham rats. In addition, MAM‐rTMS rats showed higher distance (*p* = 0.026, Bonferroni corrected) and mean speed (*p* = 0.027, Bonferroni corrected) compared to vehicle‐sham rats. There was no difference in discrimination index, distance, mean speed and central distance between MAM‐sham rats and MAM‐rTMS rats (*p* > 0.05, Bonferroni corrected; Figure S1).

### 3.2 rTMS intervention normalized frontal neuroimaging functional abnormality in MAM rats

**FIGURE 2:**
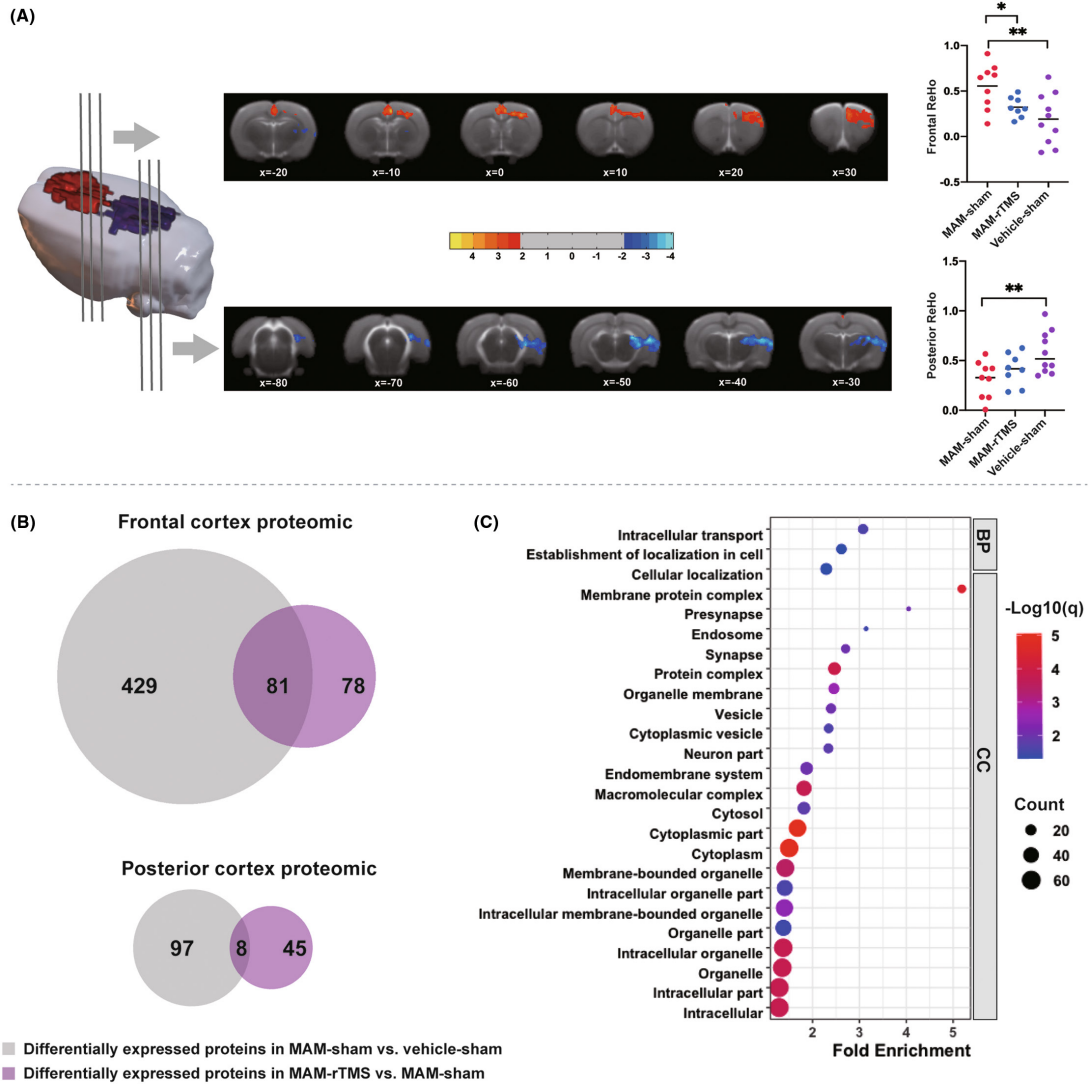
Effect of visual cortex rTMS intervention for neurodevelopmental rat model on neuroimaging and brain tissue proteomic. (A) Statistic maps showing the significant two clusters of regional homogeneity (ReHo) values between MAM‐sham group and vehicle‐sham group analyzed by whole‐brain voxel‐wise *t*‐test (all *p* < 0.05, AlphaSim corrected; cluster size ≥898). Details of the significant clusters are described in Table S2. Color bar indicates the *t* score. Red color represents the significantly increased ReHo values, and blue color represents the significantly decreased ReHo values in MAM‐sham group. On the right of Panel A, individual ReHo values of the two differential clusters were extracted and used to create the scatter plots. Statistical analysis: one‐way ANOVA with Fisher's least significant difference (LSD) for post hoc pairwise comparisons. (B) Venn diagram displaying the overlap between significantly altered proteins in MAM‐sham versus vehicle‐sham and in MAM‐rTMS versus MAM‐sham (uncorrected *p*‐value of *t*‐test <0.05 and fold change (FC) > 1.2 or <0.83). Proteins specifically altered in MAM‐sham versus vehicle‐sham are shown in gray, and MAM‐rTMS versus MAM‐sham are shown in purple respectively. There are 81 overlapping proteins in frontal cortex and 8 in posterior cortex. (C) Gene ontology (GO) enrichment analysis of 81 overlapping proteins in frontal cortex (*p* < 0.05, FDR corrected). BP, biological process terms of GO; CC, Cellular Component terms of GO. *p < 0.05, **p < 0.01.

For each rat, ReHo values at each voxel were calculated to help quantify the synchronization of local neural activity. Compared to vehicle‐sham rats, MAM‐sham rats showed significantly increased ReHo in frontal regions, including left dorsolateral orbital cortex, bilateral frontal association cortex and bilateral secondary motor cortex, and significantly decreased ReHo in posterior regions, including left primary auditory cortex, left secondary auditory cortex and left secondary somatosensory cortex. Interestingly, compared with MAM‐sham group, MAM‐rTMS group showed reduced ReHo in frontal regions, but no significant ReHo alteration in posterior regions. These results suggested that rTMS intervention with stimulation site on visual cortex might reverse frontal functional aberrance in MAM neurodevelopmental model (Figure 2A, Table S2).

### 3.3 rTMS intervention reversed frontal proteomic aberrance in MAM rats

rTMS is generally recognized as modulating the cortical protein expression to modulate neural activity.^33^ In the present study, to gain a deeper insight into the molecular mechanism of visual cortex rTMS, proteomic analyses were performed for the posterior cortex and frontal cortex, separately. A total of 3382 proteins were obtained at 1%FDR cut‐off using MaxQuant software. According to the criteria of FC > 1.2 or <0.83 and *p*‐value of *t*‐test <0.05, MAM‐sham rats had 510 aberrant protein expression in frontal cortex and 105 in posterior cortex compared to vehicle‐sham rats. And 159 different proteins were identified in frontal ROI and 53 in posterior ROI between MAM‐rTMS group and MAM‐sham group, showing the effect of rTMS on brain proteomics of MAM rats. We found 81 overlapping significant frontal proteins and 8 overlapping significant posterior proteins in both comparisons (Figure 2B, Table S4). The GO enrichment analysis of overlapping proteins in frontal ROI showed that the GO‐BP terms of intracellular transport, establishment of localization in cell, GO‐CC terms of membrane protein complex, presynapse and vesicle were significantly enriched (*p* < 0.05, FDR corrected; Figure 2C). Whereas no GO term was enriched for overlapping proteins in posterior ROI. These results showed that visual cortex rTMS intervention mainly induced neuroimaging and molecular changes in the frontal cortex in MAM model.

### 3.4 Effect of rTMS intervention in adolescent patients on clinical symptoms and neuroimaging

**FIGURE 3:**
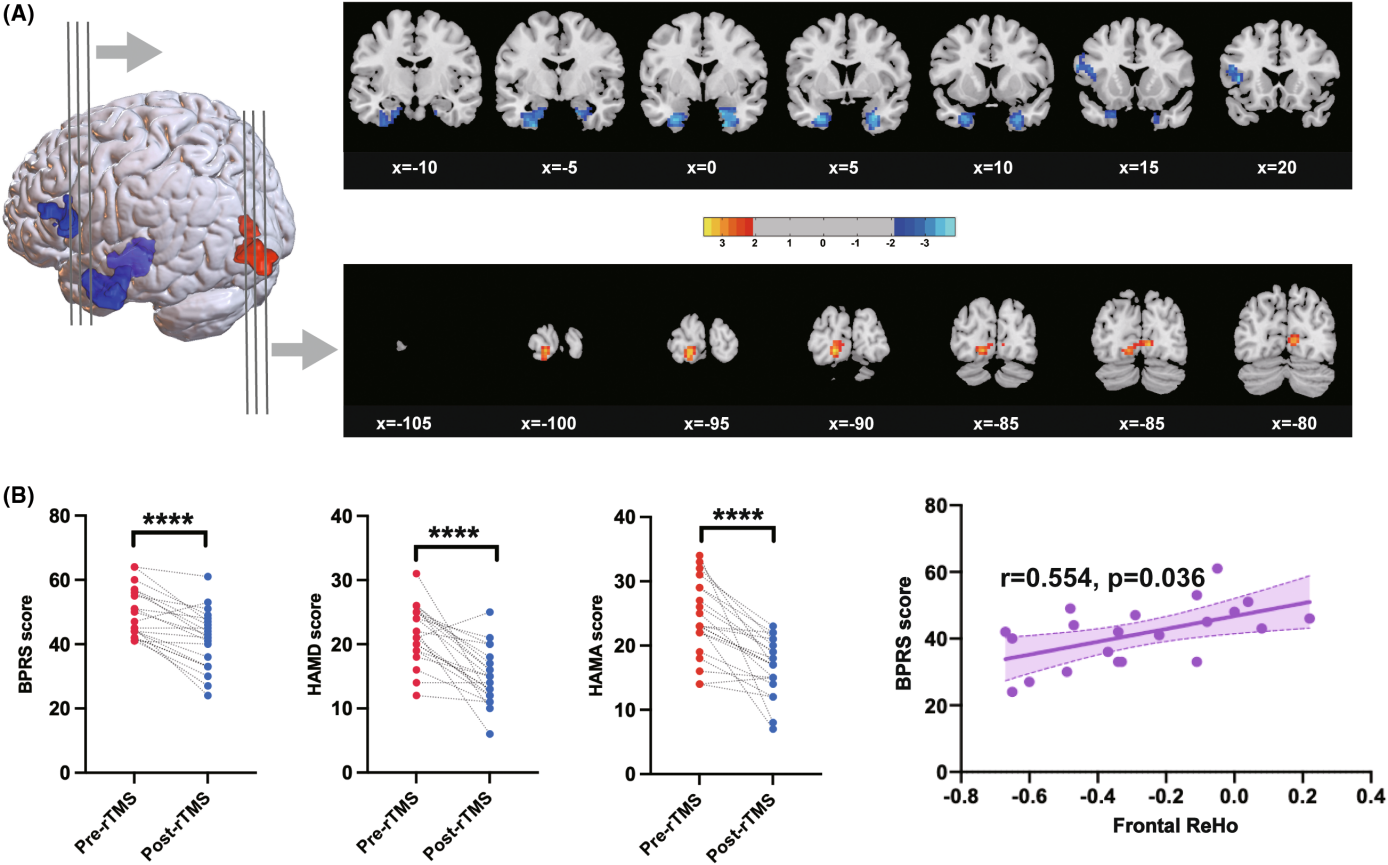
Effect of visual cortex rTMS intervention for adolescent patients with MPDs on neuroimaging and clinical symptoms. (A) Statistic maps showing regional homogeneity (ReHo) differences between pre‐ and post‐rTMS treatment analyzed by whole‐brain voxel‐wise paired *t*‐test (all *p* < 0.05, AlphaSim corrected; cluster size ≥113). Details of the significant clusters are described in Table S3. Color bar indicates the *t* score. Red color represents the significantly increased ReHo values, and blue color represents the significantly decreased ReHo values after rTMS treatment. (B) Comparison of clinical symptoms measured by BPRS, HAMD and HAMA scales between pre‐ and post‐rTMS treatment performed by paired *t*‐test. Each dot presents the score of one participant. (C) Positive correlation between the ReHo value in frontal cortex and the BPRS score after rTMS treatment (*p* < 0.05, FDR corrected). BPRS, Brief Psychiatric Rating Scale; HAMD, 17‐item Hamilton Depression Rating Scale; HAMA, Hamilton Anxiety Rating Scale. **p < 0.01, ****p < 0.0001.

To test the clinical applicability of visual cortex rTMS, adolescent patients with MPDs received 10 sessions of rTMS stimulation. To determine the effect of the treatment on the patients, ReHo values were calculated from the fMRI data and a whole‐brain voxel‐wise paired t‐test was performed for the whole brain. A decreased ReHo of frontal regions (left frontal lobe and bilateral limbic lobes) and an increased ReHo of posterior regions (left occipital cortex and left lingual gyrus) were observed after rTMS intervention (all *p* < 0.05, AlphaSim corrected; cluster size ≥113) (Figure 3A, Table S3). The results showed that rTMS produced similar neuroimaging changes in both MAM rats and adolescent patients. Furthermore, the clinical efficacy of rTMS was assessed by paired t‐test using data from clinical scales at pre‐ and post‐rTMS, which were normally distributed. Compared with pre‐rTMS, patients showed significantly reduced scores on the BPRS (*p* < 0.0001), HAMD (*p* < 0.0001) and HAMA (*p* < 0.0001) after rTMS to the visual cortex, suggesting an apparent clinical efficacy of this intervention (Figure 3B). A further positive correlation between ReHo value of left frontal lobe and BPRS score was observed after rTMS intervention (*r* = 0.554, *p* = 0.036, FDR corrected; Figure 3B), suggesting that functional changes in the frontal cortex are associated with improvement in psychiatric symptoms.

## 4 DISCUSSION

In this study, we first used a cross‐species translational approach to detect the effectiveness of visual cortex rTMS as a potential therapeutic for reversing abnormal neurodevelopment in adolescent MPDs. In the neurodevelopmental disruption model of MAM rats, a frontal‐posterior functional imbalance of increased frontal ReHo and decreased posterior ReHo was observed, representing the abnormal neurodevelopmental pattern in MPDs. visual cortex rTMS produced similar neuroimaging changes, particularly the reduction of frontal ReHo, both in the MAM rats and adolescent patients with MPDs. Furthermore, the frontal synapse‐associated proteins were found to be modulated by rTMS stimulation in the proteomic analyses, which may be the underlying molecular mechanism of rTMS efficacy. After rTMS intervention of the adolescent patients, a positive correlation was observed between frontal ReHo and the psychiatric symptom severity scaled as the BPRS score, emphasizing the key role of frontal functional neural activity in rTMS efficacy. Overall, our study highlights the frontal‐posterior functional balance and neurodevelopmental factor in the intervention of adolescent MPDs.

Neurodevelopmental disruptions have been implicated in the pathophysiology of MPDs. MAM model provides a powerful tool for understanding the neurodevelopment trajectory of MPDs. In the present study, MAM rats were observed to have significantly increased ReHo in the frontal regions and decreased ReHo in the posterior primary cortex, suggesting that frontal‐posterior functional imbalance may be the neurodevelopmental basis for MPDs. A series of our previous research using multiple neuroimaging modalities has consistently shown that increased frontal functional activity and decreased posterior functional activity are shared features across MPDs.^34^, ^35^, ^36^, ^37^ In addition, in our recent work, patients with MPDs who showed a frontal‐posterior functional imbalance had higher genetic vulnerability with increased Polygenic Risk Score for Schizophrenia and Bipolar Disorder (PRS‐SZBD) and enriched risk gene expression in brain regions including the frontal cortex, limbic system and basal ganglia.^14^ These findings support our present result, highlighting that the genetic and neurodevelopmental factors contribute to the neuroimaging pattern of frontal‐posterior imbalance.

Cortical development is known to progress from lower‐order, primary cortices with sensory and motor functions to higher‐order, transmodal association cortices supporting advanced mental functions.^12^ Our present findings show that neuromodulation of the developmental origin of primary cortex can reverse abnormal functional activity of the higher‐order regions of frontal cortex. In MAM rats, decreased ReHo was observed in the left dorsolateral orbital cortex, bilateral frontal association cortex and bilateral secondary motor cortex; and in adolescent patients, decreased ReHo was observed in the left frontal lobe and left inferior frontal gyrus. In addition, our proteomic analyses helped elucidate the molecular changes in the frontal cortex. We found that visual cortex rTMS regulated presynaptic proteins, vesicle proteins and proteins associated with intracellular transport process in the frontal brain region (Table S4). Among these, several proteins involved in neurotransmitter transport by synaptic vesicles, such as alpha‐soluble NSF attachment protein (Snap), YKT6 v‐SNARE homolog (Ykt6), actin related protein 2/3 complex, subunit 2 (Arpc2), synaptophysin‐like 1 (Sypl 1), DnaJ heat shock protein family (Hsp40) member C5 (Dnajc5) and adaptor related protein complex 2 subunit sigma 1 (Ap2s1) were found to be increased in MAM model and downregulated by rTMS.^38^, ^39^, ^40^, ^41^, ^42^, ^43^, ^44^ Neuronal calcium sensor 1(Ncs1) and rap signaling proteins can induce short‐term synaptic plasticity (STP) and long‐term potentiation (LTP),^45^, ^46^ which were observed to be increased in MAM rats and downregulated by rTMS. Our proteomic findings align well with previous research in basic neuroelectrophysiology, suggesting that rTMS‐induced changes in neuronal activity may result from the regulation of synapse‐associated proteins. In short, our cross‐species validation revealed that neuromodulation targeting primary sensory cortices (especially visual cortex) has biological efficacy on frontal cortex.

The frontal cortex functions as the “senior executive” of the brain and personality, responsible for processing, integrating, and assimilating perceptions and impulses received from the subcortices and primary cortices.^47^ Impairments in the frontal cortex have long been thought to correlate with various psychopathological symptoms.^48^ In the present study, adolescent patients with MPDs have obtained great alleviation in depressive, anxious and psychiatric symptoms as assessed by HAMD, HAMA and BPRS scales, demonstrating the clinical efficacy of visual cortex rTMS. More interestingly, it was observed that psychiatric symptoms assessed by BPRS were associated with reduced ReHo of the frontal cortex after rTMS treatment, suggesting that the clinical efficacy of rTMS may be due to biological efficacy on the frontal cortex. Similar to our findings, previous researchers have reported that visual cortex 10 HZ rTMS guided by neuroimaging biomarkers induced increased resting state functional connectivity from visual cortex to the subcortical regions of pre/subgenual anterior cingulate cortex, and further reduced clinical symptoms in MPDs.^49^ A pilot study has reported that transcranial direct current stimulation (tDCS) treatment using frontal cortex–visual occipital cortex as stimulation sites increased subcortical activity and led to a 43.8% reduction in the total score of the Montgomery‐Asberg Depression Rating Scale (MADRS), which was superior to that of tDCS using traditional stimulation sites.^50^ Therefore, the above findings revealed that noninvasive stimulation targeting the posterior primary cortex can induce activity changes of anterior subcortex and frontal cortex.

In our quest to explore the subtle frontal‐posterior imbalance, our early experiment has suggested that central carbon metabolism disturbance and mitochondrial dysfunction may underlie the ReHo alterations.^15^ Furthermore, in a prior fMRI study, we identified the nucleus accumbens as a central hub, transmitting rTMS effectiveness from posterior to frontal cortex.^51^ Additionally, a separate structural MRI study hinted at the significance of hippocampus volume reversal in neuromodulation (under revision).^52^ Our previous research has already highlighted the potential of this objective neuroimaging biomarker in guiding neurotherapeutic approaches for MPDs. To further advance our understanding, our current cross‐species study leveraged neuroimaging as a crucial “intermediate phenotype” linking precise etiological factors in MAM rats and complex symptomatologies in adolescent MPD patients, transcending species. This approach enabled us to bridge the critical translational gap from animal science to clinical practice, offering an objective approach that aligns the MPDs genomic‐proteomic‐neural‐symptomatic causal pathway with therapeutic interventions.

Despite these valuable findings, there are noteworthy limitations. We found visual cortex rTMS produced a biological effect on the frontal cortex, but the underlying neural circuit from the visual cortex to the frontal cortex remains unclear. Further circuit‐level basic experiments are warranted to enhance our understanding of the deeper biological and neural mechanisms involved in both MPDs and the effects of rTMS. In addition, the small sample size of adolescent patients in the present study may compromise the power to detect significant differences between the pre‐rTMS and post‐rTMS. Due to the lack of healthy controls, we cannot confirm that our adolescent patients showed the frontal‐posterior functional imbalance at baseline. However, in our previous work, we have found the association between such neuroimaging balance in MPDs and neurodevelopmental factor.^14^ In addition, our patients were receiving ongoing drug treatment throughout the study, we cannot rule out the influence of medication treatments on clinical outcomes. However, the effect of this confounding was minimized by ensuring stable types of medication during the rTMS trial. Given these limitations in the present study, our findings need to be validated and replicated in clinical trials with larger patient populations.

## 5 CONCLUSION

In summary, a therapeutic of visual cortex rTMS has been developed for the treatment of adolescents with MPDs and the underlying molecular mechanism of efficacy has been uncovered through this cross‐species translational study. Further mechanistic studies and clinical research are needed to validate this promising treatment.

## AUTHOR CONTRIBUTIONS

FW, RZ, XZ, JW and JL designed and conceptualized the study. FW and RZ provided supervision for the study. HG, JY, AC, and TZ participated in acquiring animal data, while JL and PZ were responsible for acquiring human patient data. JL, HG, JY, PZ, and JZ contributed to the statistical analyses. Data interpretation and manuscript preparation and revision were undertaken by JL, YX, FYW, and FW. All authors made contributions to the article and have approved the manuscript submission.

## FUNDING INFORMATION

This work was supported by the National Science Fund for Distinguished Young Scholars to FW (Grant No. 81725005); National Natural Science Foundation Regional Innovation and Development Joint Fund to FW (Grant No. U20A6005); Jiangsu Provincial Key Research and Development Program to FW (Grant No. BE2021617); National Natural Science Foundation of China to RZ (Grant No. 82151315); Jiangsu Provincial Key Research and Development Program to RZ (Grant No. BE2022160); Key Project supported by Medical Science and Technology Development Foundation, Jiangsu Commission of Health to RZ (Grant No. ZD2021026); and National Natural Science Foundation of China to XZ (Grant No. 62176129).

## CONFLICT OF INTEREST STATEMENT

The authors declare no biomedical financial interests or potential conflicts of interest.

## Supporting information


Data S1.

## Data Availability

The data that support the findings of this study are available on request from the corresponding author. The data are not publicly available due to privacy or ethical restrictions.
