# Supplementary material for: Visual cortex repetitive transcranial magnetic stimulation (rTMS) reversing neurodevelopmental impairments in adolescents with major psychiatric disorders (MPDs): A cross‐species translational study
Source: CNS Neurosci Ther. 2023 Sep 18;30(3):e14427. doi: 10.1111/cns.14427 (PMC10915985; doi:10.1111/cns.14427)
Supplement: Supplementary file 1 — Data S1. [file CNS-30-e14427-s001.docx]

**Supporting Information**

**Appendix S1. Methods**

**Animal behavioral assessment**

The novel object recognition (NOR) task, which reflects learning and recognition processing, was performed according to well-established parameters^1^*.* On day 1, all animals received a 10 min habituation session in a test box (100 × 100 × 30 cm3) with no object. On day 2, each animal was placed in the box and allowed to explore two identical objects (cylinder A and cylinder B, 6 cm in diameter, 10 cm high) horizontally inside the box (i.e., one in the northwestern corner and one in the northeastern corner) for a total of 5 min (training session-encoding phase), and exploration time was recorded during the 5 min training session. After 1 h of consolidation phase, the retention trial (testing session-retrieval phase) was conducted and one of the previously presented objects was replaced by a novel object (cone C, 6 cm in diameter, 10 cm high). All the objects were presented with similar material and size. Exploration time for the novel object during the 5 min test session was measured. Between each animal change, the cage and objects were cleaned with 70% ethanol and air-dried. Exploration time was defined as sniffing or touching the object with the nose and quantified by the camera. The NOR discrimination index was calculated from the following formula: the novel object exploration time minus the familiar object exploration time divided by the total exploration time.

The open field test (OFT) was performed in the square-shaped apparatus (100×100×30 cm3) with a video camera on top. The central 40 × 40 cm2 area was defined as the central zone. Each rat was placed in the centre of the open field, and allowed to explore freely. The movement of rat was recorded by the video camera for 5 min. The box was cleaned with 70% ethanol and air-dried after each animal. The total distance, mean speed and distance in the central area (central distance) during the 5 min of movement were measured and analyzed using video-tracking software (ANY-maze, Stoelting CO, USA)^2^.

**Animal brain tissue proteomic data acquisition and processing**

After thawing at 4°C, each brain tissue sample (60mg) was added to 100μl of SDT lysis buffer (4%SDS, 100mM Tris-HCl, 1mM DTT, pH7.6). The mixture was sonicated for 1 min following homogenization (24×2, 6.0M/S, 60s, twice), incubated in boiling water for 15 min, and then centrifuged at 14000g for 40 min. The supernatant was transferred to filters (0.22µm), and the protein concentration was determined using the BCA Protein Assay Kit (Bio-Rad, USA). Each sample (200μg) was then dialyzed and reduced with 30μl SDT buffer (4% SDS, 100mM DTT, 150mM Tris-HCl, pH 8.0). UA buffer (8M Urea, 150mM Tris-HCl, pH 8.0) was added to remove DTT and other low-molecular-weight components by repeated ultrafiltration (Microcon units, 10kD). Then 100μl of iodoacetamide (100mM IAA in UA buffer) was added to block reduced cysteine residues. After three washes with 100μl UA buffer and two washes with 100μl 25mM NH4HCO3 buffer twice, the samples were incubated overnight at 37°C with 4μg trypsin (Promega) in 40μl 25mM NH4HCO3 buffer. Desalting of the protein digests was performed on C18 Cartridges (Empore™ SPE Cartridges C18, bed I.D. 7mm, volume 3ml, Sigma). Finally, the peptides were reconstituted in 40µl of 0.1% (v/v) formic acid for subsequent LC-MS/MS analysis.

Proteomic acquisition was achieved on a Q Exactive mass spectrometer coupled with Easy nanoLC (Thermo Fisher Scientific). Peptides were injected onto a reversed-phase trap column (Thermo Scientific Acclaim PepMap100, 100μm*2cm, nanoViper C18) connected to the C18 reversed-phase analytical column (Thermo Scientific Easy Column, 10cm long, 75μm inner diameter, 3μm resin) in buffer A (0.1% formic acid) and eluted using a linear gradient of buffer B (84% acetonitrile and 0.1% formic acid) at a constant flow rate of 300 nl/min controlled by intelliflow technology. The 2-hour gradient started with 110 min at 0-55% buffer B, followed by a 5 min at 55-100% buffer B, and then 10 min equilibration in 100% buffer B. The mass spectrometer was operated in positive ion mode and the 10 most abundant parent ions from the survey scan were selected for fragmentation via higher energy collision induced dissociation (HCD). Other parameters for MS scan were set as follows: the dynamic exclusion time was 25s; the automatic gain control (AGC) target was set to 3e6 with a maximum injection time of 10ms; the MS survey scans were acquired from a mass range of 300-1800m/z with a resolution of 70000 at m/z 200; the resolution was set to 17500 at m/z 200 for HCD spectra. The normalized collision energy was 30 eV; the underfill ratio was set to 0.1%.

The MS data generated from proteomic acquisition were preprocessed using MaxQuant software version 1.3.0.5 (Max Planck Institute of Biochemistry in Martinsried, Germany). Search parameters were as follows: Enzyme: trypsin; Max missed cleavages: two; Main search: 6ppm; First search: 20ppm; MS/MS tolerance: 20 ppm; Fixed modifications: carbamidomethyl (C); Variable modifications: oxidation (M) and acetyl (protein N-term); Database: ensembl_rattus_29107_20190628.fasta, uniprot_mouse_76417_20141212.fasta, and a self-built database created using authentic standards; Database pattern: reverse. Label-free quantification was carried out in MaxQuant using intensity determination and normalization algorithm^3^. Protein abundance was measured from the normalized spectral protein intensity (LFQ intensity). The MS results were forwarded to statistical analysis.

**References:**

1. Grayson B, Leger M, Piercy C, Adamson L, Harte M, Neill J. Assessment of disease-related cognitive impairments using the novel object recognition (NOR) task in rodents. *Behavioural brain research.* 2015;285:176-193.

2. Nozari M, Mansouri FA, Shabani M, Nozari H, Atapour N. Postnatal MK-801 treatment of female rats impairs acquisition of working memory, but not reference memory in an eight-arm radial maze; no beneficial effects of enriched environment. *Psychopharmacology.* 2015;232(14):2541-2550.

3. Cox J, Hein M, Luber C, Paron I, Nagaraj N, Mann M. Accurate proteome-wide label-free quantification by delayed normalization and maximal peptide ratio extraction, termed MaxLFQ. *Molecular & cellular proteomics : MCP.* 2014;13(9):2513-2526.

**Figure S1.** Results of the discrimination index (DI) (A) of the novel object recognition (NOR) task, distance (B), mean speed (C) and central distance (D) of the open field test (OFT). Statistical analysis: one-way ANOVA with Bonferroni test for pos-hoc analysis. * represents P < 0.05, ** represents P < 0.01.

D\D

C

B

A

Central distance

DI

Distance

Mean speed

**Table S1.** Participant demographics and baseline characteristics.

| Participants | N=21 |
| --- | --- |
| Diagnosis, N (%) |  |
| SZ | 2 (9.52%) |
| BD | 7 (33.33%) |
| MDD | 12 (57.14%) |
| Age, mean (SD) | 16 .28 (0.95) |
| Female, N (%) | 12 (57.14%) |
| BMI, mean (SD) | 23.28 (5.32) |
| Han race, N (%) | 21 (100%) |
| Education years, mean (SD) | 10.39 (1.06) |
| Right handedness, N (%) | 15 (71%) |
| First episode, N (%) | 13 (61.90%) |
| Disease duration, month, mean (SD) | 14.74 (3.19) |
| Baseline scale scores, mean (SD) |  |
| BPRS | 49.62 (7.65) |
| HAMD | 21.71 (4.51) |
| HAMA | 24.10 (5.90) |
| Medications, N (%) |  |
| Antidepressant | 3 (14.29%) |
| Antipsychotic | 20 (95.24%) |
| Mood stabilizer | 17 (80.95%) |
| Sedative hypnotic | 3 (14.29%) |

SZ, schizophrenia; BD, bipolar disorder; MDD, major depressive disorder; SD, standard deviation; BPRS, the Brief Psychiatric Rating Scale; HAMD, the Hamilton Depression Rating Scale 17-items; HAMA, the Hamilton Anxiety Rating Scale

**Table S2.** Overlapping brain areas between regions of Interest (ROIs) and the significant clusters identified between MAM-sham group and vehicle-sham group.

| **Brain region** | **Paxinos-Watson**  **brain atlas** |  | **Number of voxels** |
| --- | --- | --- | --- |
| **Cluster 1 (Frontal)** |  |  | 984 |
| Left dorsolateral orbital cortex | 266 |  |  |
| Bilateral frontal association cortex | 333 |  |  |
| Bilateral secondary motor cortex | 507 |  |  |
| **Cluster 2 (Posterior)** |  |  | 223 |
| Left primary auditory cortex | 145 |  |  |
| Left secondary auditory cortex | 146 |  |  |
| Left secondary somatosensory cortex | 760 |  |  |

**Table S3.** Details of the four significant clusters identified between pre- and post-rTMS treatment.

| **Brain region** | **Peak MNI coordinate** | | | **Number of voxels** |
| --- | --- | --- | --- | --- |
| **Cluster 1** | -51 | 18 | 18 | 126 |
| Left cerebrum |  |  |  | 126 |
| Frontal lobe |  |  |  | 125 |
| Inferior frontal gyrus |  |  |  | 98 |
| **Cluster 2** | 6 | -78 | 3 | 160 |
| Left cerebrum |  |  |  | 157 |
| Occipital lobe |  |  |  | 121 |
| Lingual gyrus |  |  |  | 109 |
| **Cluster 3** | 30 | 6 | -33 | 194 |
| Right cerebrum |  |  |  | 181 |
| Limbic lobe |  |  |  | 102 |
| Uncus |  |  |  | 80 |
| **Cluster 4** | -30 | 3 | -36 | 241 |
| Left cerebrum |  |  |  | 220 |
| Limbic lobe |  |  |  | 127 |
| Uncus |  |  |  | 104 |

**Table S4.** 81 overlapping differential proteins of frontal cortex in pairwise comparisons of MAM-sham group and vehicle-sham group, and of MAM-rTMS group and MAM-sham group.

| **Protein Name** | **Gene Name** | **MAM-sham vs. Vehicle-sham** | |  | **MAM-rTMS vs. MAM-sham** | |
| --- | --- | --- | --- | --- | --- | --- |
|  |  | **Fold Change ^a^** | **P Value** |  | **Fold Change ^a^** | **P Value** |
| synaptophysin-like 1 | Sypl1 | 1.826 | 0.005 |  | 0.669 | 0.023 |
| RAP2B, member of RAS oncogene family | Rap2b | 1.735 | 0.011 |  | 0.611 | 0.003 |
| NADH:ubiquinone oxidoreductase subunit A8 | Ndufa8 | 1.674 | 0.001 |  | 0.746 | 0.022 |
| G protein subunit gamma 2 | Gng2 | 1.612 | 0.035 |  | 0.502 | 0.013 |
| eukaryotic translation initiation factor 5A | Eif5a | 1.606 | 0.005 |  | 0.709 | 0.018 |
| RAP1B, member of RAS oncogene family | Rap1b | 1.603 | 0.001 |  | 0.751 | 0.023 |
| neuronal calcium sensor 1 | Ncs1 | 1.592 | 0.034 |  | 0.690 | 0.049 |
| cytochrome c oxidase subunit 7C | Cox7c | 1.554 | 0.019 |  | 0.566 | 0.013 |
| actin related protein 2/3 complex, subunit 2 | Arpc2 | 1.514 | 0.000 |  | 0.784 | 0.006 |
| diacylglycerol kinase, theta | Dgkq | 1.495 | 0.015 |  | 0.673 | 0.019 |
| DnaJ heat shock protein family (Hsp40) member C5 | Dnajc5 | 1.468 | 0.008 |  | 0.727 | 0.016 |
| voltage-dependent anion channel 3 | Vdac3 | 1.467 | 0.007 |  | 0.742 | 0.017 |
| SPG7, paraplegin matrix AAA peptidase subunit | Spg7 | 1.421 | 0.040 |  | 0.633 | 0.035 |
| adaptor related protein complex 2 subunit sigma 1 | Ap2s1 | 1.414 | 0.017 |  | 0.737 | 0.032 |
| Ras-related GTP binding A | Rraga | 1.409 | 0.012 |  | 0.581 | 0.014 |
| ArfGAP with GTPase domain, ankyrin repeat and PH domain 3 | Agap3 | 1.400 | 0.002 |  | 0.827 | 0.047 |
| YKT6 v-SNARE homolog | Ykt6 | 1.384 | 0.007 |  | 0.788 | 0.021 |
| ADAM metallopeptidase domain 23 | Adam23 | 1.364 | 0.019 |  | 0.728 | 0.025 |
| flotillin 1 | Flot1 | 1.362 | 0.001 |  | 0.747 | 0.008 |
| tyrosine 3-monooxygenase/tryptophan 5-monooxygenase activat | Ywhaq | 1.356 | 0.002 |  | 0.781 | 0.006 |
| secretory carrier membrane protein 5 | Scamp5 | 1.355 | 0.011 |  | 0.688 | 0.038 |
| translocase of inner mitochondrial membrane 50 | Timm50 | 1.351 | 0.002 |  | 0.830 | 0.024 |
| platelet-activating factor acetylhydrolase 1b, regulato | Pafah1b1 | 1.344 | 0.005 |  | 0.789 | 0.015 |
| glutathione S-transferase mu 2 | Gstm2 | 1.309 | 0.011 |  | 0.762 | 0.016 |
| VPS26 retromer complex component B | Vps26b | 1.301 | 0.001 |  | 0.827 | 0.015 |
| VPS35 retromer complex component | Vps35 | 1.284 | 0.022 |  | 0.792 | 0.048 |
| Coatomer subunit beta' | Copb2 | 1.288 | 0.035 |  | 0.677 | 0.001 |
| RAP1A, member of RAS oncogene family | Rap1a | 1.271 | 0.027 |  | 0.773 | 0.025 |
| NSF attachment protein alpha | Napa | 1.215 | 0.024 |  | 0.804 | 0.021 |
| actin-related protein 10 homolog | Actr10 | 0.760 | 0.032 |  | 0.750 | 0.004 |
| ATPase H+ transporting V1 subunit F | Atp6v1f | 0.754 | 0.024 |  | 1.333 | 0.039 |
| coiled-coil-helix-coiled-coil-helix domain containing 6 | Chchd6 | 0.604 | 0.000 |  | 1.611 | 0.010 |
| prostaglandin D2 synthase | Ptgds | 0.590 | 0.026 |  | 2.069 | 0.026 |
| G protein-coupled receptor 37-like 1 | Gpr37l1 | 2.173 | 0.000 |  | 0.706 | 0.008 |
| hippocalcin-like 1 | Hpcal1 | 2.163 | 0.033 |  | 0.354 | 0.011 |
| pyridine nucleotide-disulphide oxidoreductase domain 2 | Pyroxd2 | 1.999 | 0.002 |  | 0.644 | 0.023 |
| fatty acid binding protein 5 | Fabp5 | 1.907 | 0.025 |  | 0.518 | 0.025 |
| Pirin | Pir | 1.787 | 0.002 |  | 0.697 | 0.043 |
| Translin | Tsn | 1.770 | 0.041 |  | 0.515 | 0.034 |
| BMP/retinoic acid inducible neural specific 1 | Brinp1 | 1.733 | 0.000 |  | 0.759 | 0.014 |
| transcription elongation factor A like 3 | Tceal3 | 1.718 | 0.000 |  | 0.741 | 0.011 |
| solute carrier family 22, member 23 | Slc22a23 | 1.599 | 0.000 |  | 0.738 | 0.004 |
| baculoviral IAP repeat-containing 6 | Birc6 | 1.558 | 0.011 |  | 0.686 | 0.012 |
| receptor accessory protein 1 | Reep1 | 1.531 | 0.004 |  | 0.714 | 0.006 |
| carboxymethylenebutenolidase homolog | Cmbl | 1.504 | 0.038 |  | 0.663 | 0.034 |
| proteasome 26S subunit, ATPase 1 | Psmc1 | 1.496 | 0.000 |  | 0.830 | 0.014 |
| ARP1 actin related protein 1 homolog A | Actr1a | 1.424 | 0.016 |  | 0.666 | 0.008 |
| OTU deubiquitinase, ubiquitin aldehyde binding 1 | Otub1 | 1.403 | 0.003 |  | 0.764 | 0.009 |
| Rho GTPase activating protein 35 | Arhgap35 | 1.376 | 0.017 |  | 0.650 | 0.023 |
| YTH N(6)-methyladenosine RNA binding protein 3 | Ythdf3 | 1.369 | 0.002 |  | 1.244 | 0.019 |
| La ribonucleoprotein domain family, member 1 | Larp1 | 1.353 | 0.010 |  | 0.789 | 0.006 |
| histidyl-tRNA synthetase | Hars | 1.351 | 0.001 |  | 0.823 | 0.044 |
| thioesterase superfamily member 4 | Them4 | 1.332 | 0.001 |  | 0.795 | 0.014 |
| actin related protein 2/3 complex, subunit 1A | Arpc1a | 1.319 | 0.024 |  | 0.740 | 0.039 |
| SUB1 homolog, transcriptional regulator | Sub1 | 1.310 | 0.005 |  | 0.761 | 0.005 |
| exocyst complex component 2-like | LOC103689971 | 1.298 | 0.003 |  | 0.800 | 0.025 |
| similar to RIKEN cDNA D230025D16Rik | RGD621098 | 1.284 | 0.046 |  | 0.714 | 0.013 |
| methylosome protein 50-like | LOC100911453 | 1.267 | 0.006 |  | 0.796 | 0.023 |
| proliferation-associated 2G4 | Pa2g4 | 1.267 | 0.002 |  | 0.826 | 0.026 |
| elongator acetyltransferase complex subunit 2 | Elp2 | 1.260 | 0.013 |  | 0.773 | 0.017 |
| elongin B | Elob | 1.253 | 0.000 |  | 0.822 | 0.002 |
| phosphatidylinositol transfer protein, alpha | Pitpna | 1.249 | 0.014 |  | 0.805 | 0.034 |
| CDGSH iron sulfur domain 1 | Cisd1 | 1.243 | 0.016 |  | 0.822 | 0.009 |
| leucine rich repeat containing 59 | Lrrc59 | 0.808 | 0.018 |  | 1.225 | 0.028 |
| selenoprotein I | Selenoi | 0.796 | 0.008 |  | 1.267 | 0.010 |
| 40S ribosomal protein S29 | LOC108352650 | 0.785 | 0.039 |  | 1.628 | 0.018 |
| DnaJ heat shock protein family (Hsp40) member B6 | Dnajb6 | 0.778 | 0.019 |  | 1.347 | 0.015 |
| glycine cleavage system protein H | Gcsh | 0.776 | 0.004 |  | 1.233 | 0.031 |
| ribosomal protein S5 | Rps5 | 0.747 | 0.008 |  | 1.405 | 0.010 |
| adipocyte plasma membrane associated protein | Apmap | 0.744 | 0.009 |  | 1.278 | 0.045 |
| host cell factor C1 | Hcfc1 | 0.734 | 0.011 |  | 1.252 | 0.043 |
| ArfGAP with GTPase domain, ankyrin repeat and PH domain 1 | Agap1 | 0.716 | 0.034 |  | 1.450 | 0.047 |
| apoptotic chromatin condensation inducer 1 | Acin1 | 0.711 | 0.009 |  | 1.287 | 0.041 |
| protein phosphatase 2, regulatory subunit B, beta | Ppp2r5b | 0.704 | 0.006 |  | 1.305 | 0.035 |
| small nuclear ribonucleoprotein polypeptide G | Snrpg | 0.663 | 0.008 |  | 1.322 | 0.014 |
| hyaluronan binding protein 4 | Habp4 | 0.661 | 0.014 |  | 1.424 | 0.045 |
| Phosphatidylinositol 5-phosphate 4-kinase type-2 alpha | Pip4k2a | 0.659 | 0.006 |  | 1.268 | 0.024 |
| MAP7 domain containing 2 | Map7d2 | 0.620 | 0.013 |  | 1.407 | 0.026 |
| RBMX like 1 | Rbmxl1 | 0.496 | 0.003 |  | 2.019 | 0.007 |
| RNA binding motif protein 5 | Rbm5 | 0.463 | 0.003 |  | 1.836 | 0.012 |
| hemoglobin alpha, adult chain 2 | Hba-a2 | 0.449 | 0.014 |  | 1.841 | 0.031 |

^a^ In MAM-sham vs. vehicle-sham, fold change more than 1 representing higher levels in MAM-sham and fold change less than 1 representing lower levels in MAM-sham. Similarly, in MAM-rTMS vs. MAM-sham, fold change more than 1 representing higher levels in MAM-rTMS and fold change less than 1 representing lower levels in MAM-rTMS.
